# Hybrid polymer/ZnO solar cells sensitized by PbS quantum dots

**DOI:** 10.1186/1556-276X-7-106

**Published:** 2012-02-07

**Authors:** Lidan Wang, Dongxu Zhao, Zisheng Su, Dezhen Shen

**Affiliations:** 1 State Key Laboratory of Luminescence and Applications, Changchun Institute of Optics, Fine Mechanics and Physics, Chinese Academy of Sciences, Changchun 130033, People's Republic of China; 2 Graduate School of Chinese Academy of Sciences, Beijing 100039, People's Republic of China

## Abstract

Poly[2-methoxy-5-(2-ethylhexyloxy-*p-*phenylenevinylene)]/ZnO nanorod hybrid solar cells consisting of PbS quantum dots [QDs] prepared by a chemical bath deposition method were fabricated. An optimum coating of the QDs on the ZnO nanorods could strongly improve the performance of the solar cells. A maximum power conversion efficiency of 0.42% was achieved for the PbS QDs' sensitive solar cell coated by 4 cycles, which was increased almost five times compared with the solar cell without using PbS QDs. The improved efficiency is attributed to the cascade structure formed by the PbS QD coating, which results in enhanced open-circuit voltage and exciton dissociation efficiency.

## Introduction

Hybrid polymer solar cell is a promising photovoltaic technology, offering environmental stability, low-cost manufacturing, and versatile applicability [1-3]. The solution processing of polymer organic photovoltaic devices may offer an inexpensive technology to fabricate solar cells with large areas. Hybrid polymer-inorganic solar cells utilize the high electron mobility inorganic phase to overcome charge-transport limitations associated with organic materials. Zinc oxide [ZnO] has been regarded as an excellent semiconductor material for the solar cell due to its high electron mobility as well as the high chemical and thermal stability [4,5]. Compared with ZnO bulk materials, one-dimensional nanostructures have some special advantages for optoelectronic devices including the large surface area to significantly increase the junction area, the enhanced polarization dependence, and the improved carrier confinement in one dimension. Various polymer/ZnO hybrid solar cells have been reported [1,6]. However, the power conversion efficiencies [*η*_P_] of these devices are still low and need to be further enhanced [4,7].

Pursuing high efficiency is indeed a core task for hybrid solar cell systems, and one of the current key issues is to search the suitable panchromatic sensitizers for enhancing the light harvest under a visible light region. In addition to traditional dye sensitizers, semiconductor quantum dots [QDs] have been researched as possible alternative sensitizers due to their high expectation of having the following advantages over molecular dyes: (1) facile tuning of effective bandgaps down to the infrared [IR] range by changing their sizes and compositions, (2) higher stability and resistivity toward oxygen and water over molecular counterparts, (3) new possibilities for making multilayer or hybrid sensitizers, and (4) new phenomena such as multiple exciton generation and use of energy transfer-based charge collection as well as direct charge transfer schemes. By now, many experiments have proved that it is possible to utilize hot electrons to generate multiple electron-hole pairs per photon through the impact ionization effect by using QDs [3]. The concept of QD sensitization has been considered to be of great promise in increasing the *η*_P _of the organic/inorganic hybrid solar cells. Various semiconductor QDs such as CdS [8-10], CdSe [11,12], PbS [13,14], PbSe [15,16], and InP [17] have been adopted in the hybrid solar cells. The QD-sensitized ZnO nanorod-based liquid solar cells have been proposed, but few QD-sensitized organic/ZnO nanorod hybrid solar cells sensitized by PbS QDs have been reported in the literature. The possible reasons may be that the QDs synthesized by the conventional solution methods have some surfactant molecules on the surface, which may block the transfer of the photogenerated carriers in QDs, and the preparation of PbS QDs on the ZnO nanorods was extremely difficult due to the high acidity of the lead salt. In this paper, we designed a hybrid ZnO nanorod solar cell, in which a ZnO nanorod array with a diameter of 40 to 80 nm and a length of 200 to 300 nm served as the n-type semiconductor and poly[2-methoxy-5-(2-ethylhexyloxy-*p*-phenylenevinylene)] [MEH-PPV] was adopted as the hole transfer layer. A thin PbS QD layer was sandwiched between ZnO and MEH-PPV layers synthesized by the chemical bath deposition [CBD] method. When PbS was introduced as the QD sensitizer, a cascade energy alignment was formed in the hybrid solar cell, and a *η*_P _as high as 0.42% was achieved.

## Experimental section

Among the various techniques to grow one-dimensional ZnO nanostructures, the cost-effective electrodeposition [18] method was used in this work for the nanorod preparation with large areas because of the low-temperature processing, arbitrary substrate shapes, and precise control of the size of nanorods. Firstly, a seed layer of 30 nm was grown by RF magnetron sputtering on cleaned indium tin oxide [ITO]-coated glass substrates with a sheet resistance of 25 Ω/sq. Then, ZnO nanorods were electrodeposited in 0.005 M Zn(NO_3_)_2 _and 0.005 M hexamethylenetramine aqueous solutions. All depositions were carried out in a configured glass cell at 90°C, in which the ITO substrate, a platinum plate, and an Ag/AgCl electrode in a saturated KCl solution served as the working electrode, the counter electrode, and the reference electrode, respectively. All electrodepositions were done at a potential of -0.9 V vs. the reference electrode. The durations of the deposition were 20 min. The PbS quantum dots were deposited by the simple CBD method. The CBD process involved dipping the prepared ZnO nanorod array substrate in a methanol solution consisting of 0.01 M lead acetate for 5 min and dipping it in another methanol solution consisting of 0.005 M Na_2_S for 10 min. After each step, the substrate was rinsed with methanol. The two-step dipping procedure was considered one CBD cycle. The amount of PbS can be increased by repeating the cycles. Subsequently, the samples were thoroughly washed with deionized water and then dried at room temperature. MEH-PPV in chloroform (20 mg/ml) was spin-coated onto the surface of the PbS QD/ZnO nanorod structures at 2,000 r/min. Films were baked in a vacuum oven for 30 min at 100°C. Then, a thin layer of poly(3,4-ethylenedioxythiophene)-poly(styrenesulfonate) (PEDOT:PSS) was spin-coated on the MEH-PPV film at 2,000 r/min and baked in a vacuum oven for 1 h at 120°C. Finally, Au was evaporated onto the device as the top electrode. The field emission scanning electron microscopy [FESEM] measurements were performed by the Hitachi FESEM S-4800 (Hitachi, Ltd., Chiyoda, Tokyo, Japan). The absorption spectrum was recorded using a Shimadzu UV-3101PC spectrophotometer (Shimadzu Corporation, Nakagyo-ku, Kyoto, Japan). Current-voltage [*I*-*V*] characteristics of the devices were measured using a Keithley 2400 SourceMeter (Keithley Instruments Inc., Cleveland, OH, USA) connected with a GPIB controller to a computer under dark or one sun illumination (AM1.5, 100 mW/cm^2^). All the measurements were carried out at room temperature under ambient conditions.

## Results and discussion

**Figure 1:**
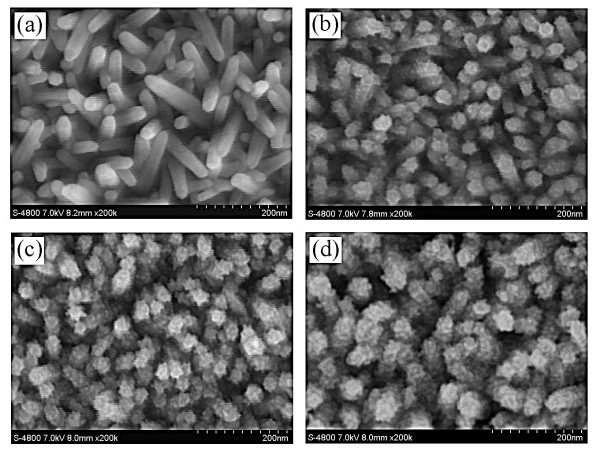
**FESEM image of as-prepared ZnO nanorods and PbS QD-coated ZnO nanorod arrays with different CBD cycles**. (**a**) ZnO nanorods, (**b**) ZnO nanorods coated by PbS QDs with 2 CBD cycles, (**c**) ZnO nanorods coated by PbS QDs with 4 CBD cycles, and (**d**) ZnO nanorods coated by PbS QDs with 6 CBD cycles. The scale bar is 200 nm.

Figure 1 shows the typical FESEM images of ZnO nanorod arrays on an ITO glass substrate and PbS QD-coated ZnO nanorod arrays with different CBD cycles. It can be observed that the diameters of the PbS QDs are enlarged with the increasing CBD cycles. The surface of the ZnO nanorods was partially coated with the PbS QDs (Figure 1b,c) with 2 and 4 CBD cycles. However, almost the whole surface of the ZnO nanorods was coated with the PbS QDs (Figure 1d) with 6 CBD cycles, and the PbS QDs were arranged closely to each other.

**Figure 2:**
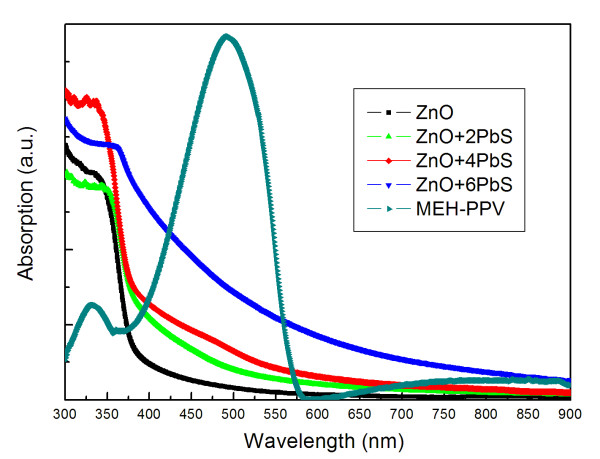
**Absorption spectrum of ZnO nanorods, ZnO nanorods with different cycles of CBD-PbS QDs, and MEH-PPV**.

Figure 2 shows the absorption spectra of ZnO nanorods, PbS QD-coated ZnO nanorod arrays with different CBD cycles, and MEH-PPV. As shown in the figure, the ZnO nanorods only absorb the high energy light with a wavelength shorter than 370 nm. With the PbS coating, the UV optical absorption edges of ZnO/PbS hybrid nanostructures are red-shifted to the long-wavelength side gradually with increasing CBD growth cycles. The absorption for the visible region is also increased a lot, ascribing to the narrow bandgap of PbS. A strong increase in absorption after each step can be observed, suggesting an increase in the number of quantum dots as well as a bathochromic spectral shift. This effect can be explained by an increase in the size after each deposition step in terms of the size quantization effect. On the other hand, the MEH-PPV showed a predominant absorption band at 400 to 570 nm.

**Figure 3:**
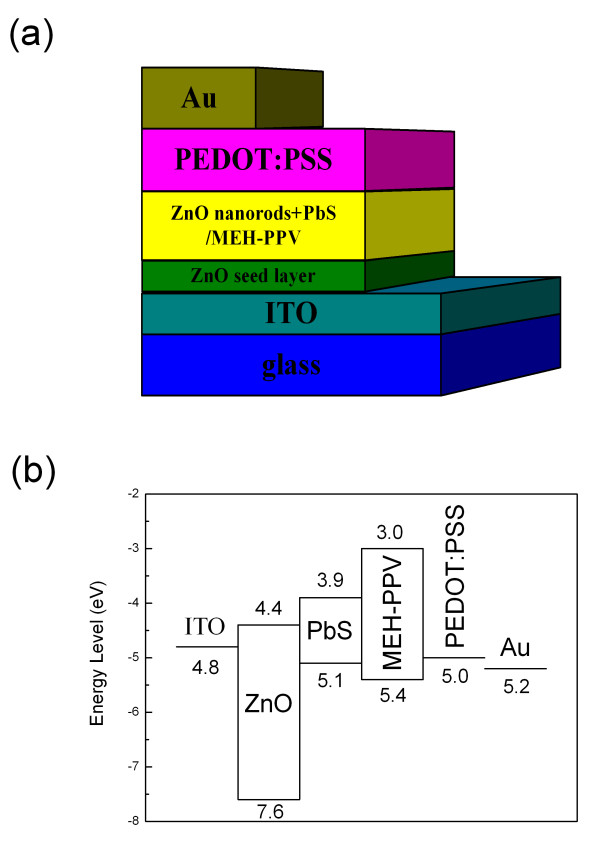
**Device structure (a) and band diagram (b) for polymer/ZnO solar cell sensitized by PbS QDs**.

**Figure 4:**
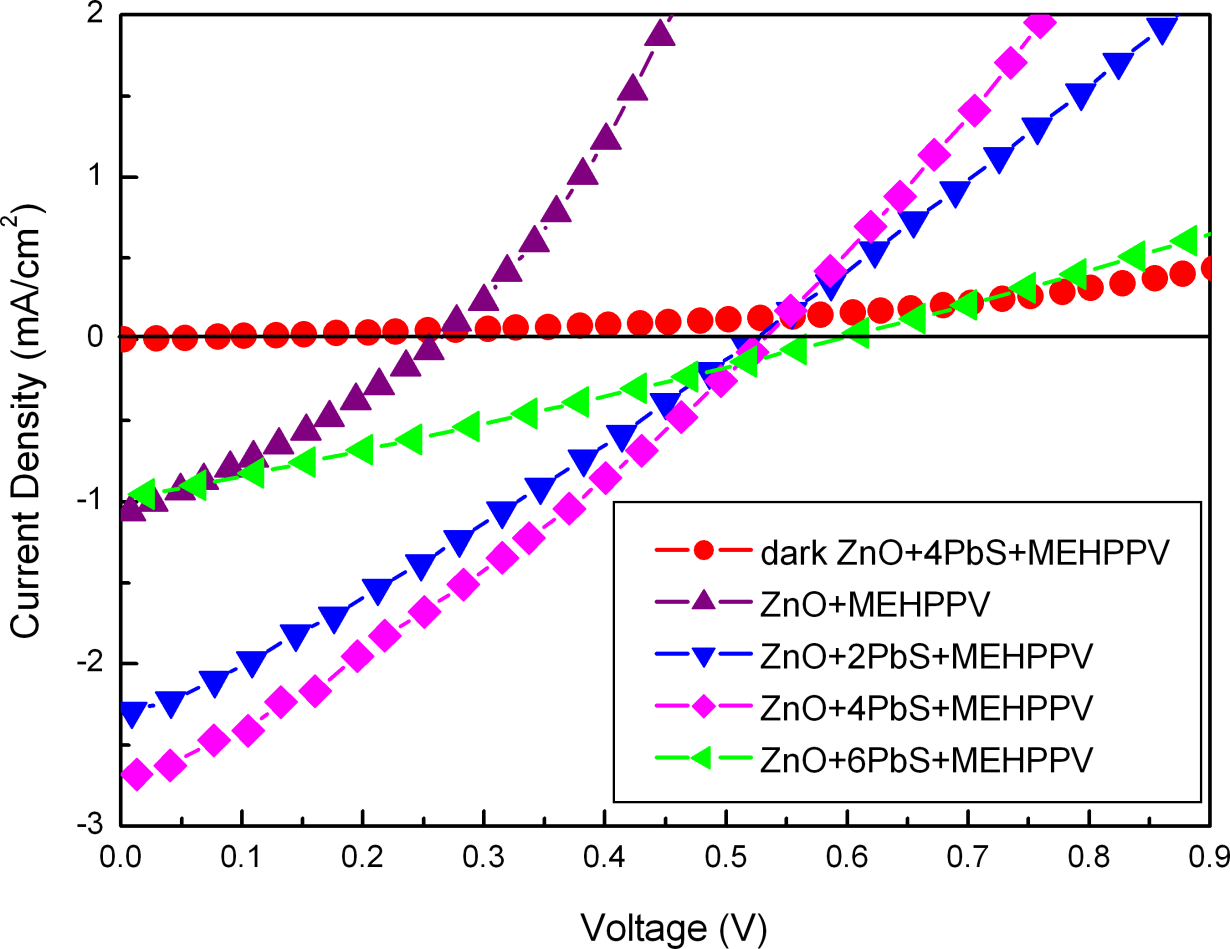
***I*-*V *characteristics**. The *I*-*V *characteristics of the polymer/ZnO solar cell and the solar cells sensitized by the PbS quantum dots under an illumination of 1.5 sun (100 mW cm^-2^) and the *I*-*V *characteristics of the solar cell after introduction of PbS quantum dots using 4 CBD cycles in the dark.

The device structure and band diagram of the PbS [19] QD-sensitized MEH-PPV/ZnO [20] solar cells with a cascade energy alignment were shown in Figure 3. Figure 4 shows the *I*-*V *characteristics of the MEH-PPV/ZnO solar cell and the solar cells sensitized by the PbS QDs under one sun illumination (AM1.5, 100 mW/cm^2^) and the dark current of the PbS QD-sensitized solar cell with 4 CBD cycles. Detail parameters of the solar cells extracted from the *I*-*V *characteristics were listed in Table 1. The MEH-PPV/ZnO solar cell shows a short-circuit current density [*J*_SC_], an open-circuit voltage [*V*_OC_], a fill factor [FF], and a *η*_P _of 1.06 mA/cm^2^, 0.25 V, 0.30, and 0.09%, respectively. The *J*_SC _of the PbS QD-sensitized solar cells increased with the CBD cycles, and a maximum *J*_SC _of 2.68 mA/cm^2 ^was obtained with 4 CBD cycles. The enhancement of the *J*_SC _in the device should be due to the enhancement in absorption of the increasing PbS in Figure 2[14,21]. Meanwhile, the *V*_OC _of the PbS QD-sensitized solar cells increases monotonically with the CBD cycles, and a maximum *V*_OC _of 0.59 V is found with 6 CBD cycles. However, the FF did not show much difference with different CBD cycles. As a result, the PbS QD-sensitized solar cell with 4 CBD cycles shows a maximum *η*_P _of 0.42%, which was increased almost five times compared with the one without PbS QDs.

**Table 1:** Parameters of polymer/ZnO solar cells sensitized by various PbS quantum dots

CBD cycle	*J*_SC_ (mA/cm^2^)	*V*_OC_ (V)	FF	*η*_P_ (%)
0	1.06	0.25	0.30	0.09
2	2.28	0.51	0.30	0.34
4	2.68	0.55	0.29	0.42
6	0.76	0.59	0.28	0.16

CBD, chemical bath deposition; *J*_SC_, short-circuit current density; *V*_OC_, open-circuit voltage; FF, fill factor; *η*_P_, power conversion efficiency.

The *V*_OC _was reported to track the energy difference between the highest occupied molecular orbital level of the donor and the lowest unoccupied molecular orbital level of the conduction band edge of the acceptor [5,22]. From the band diagram in Figure 3, the *V*_OC _increase in the PbS QD-sensitized MEH-PPV/ZnO solar cells can be reasonably understood because the conduction band of PbS is higher (lower electron affinity) than that of ZnO. Besides, the passivation of the surface states of the ZnO nanorods by the PbS coating led to the decreased recombination or charge trapping, and the cascade structure formed a charge carrier recombination barrier which could result in *V*_OC _improvement.

By measuring devices with different cycles of coatings, it can be seen that an optimum performance of the cell exists. The enhancement of *J*_SC _can be attributed to the cascade band structure formed with the PbS QD coating and to the higher carrier mobility in inorganic semiconductors. Such a cascade structure ensures that excitons formed in any of the three materials, e.g., ZnO nanorods, PbS QDs, and MEH-PPV, could be dissociated into a free electron and hole at the ZnO/PbS and PbS/MEH-PPV interfaces. Then, the electrons and holes will transport through ZnO and MEH-PPV to the ITO and Au electrodes, respectively. The cascade structure will restrict electron and hole recombination when transporting in the active layers and hence leads to a high charge carrier extraction efficiency. Moreover, the ZnO seed layer would avoid the direct contact between MEH-PPV and the ITO electrode and forbid the hole leakage to the ITO electrode. These factors, combining with the high *V*_OC_, bring a high *η*_P _of 0.42% in the 4 CBD cycles of the PbS QD-sensitized MEH-PPV/ZnO solar cell. When the CBD cycles further increases, *J*_SC _decreases. The decrease of *J*_SC _with the increasing CBD cycles is considerably attributed to two reasons: one is the decrease of ZnO amount due to the growth of PbS QDs which is an erosive process [23] for the ZnO nanorods, and the other is ascribed to the interface restriction for the carrier transfer process between QDs. With increasing quantum of QDs, the resistance originating from the interface would become more and more dominant in the devices. For large clusters, the band alignment at the ZnO/PbS interface appears to be unfavorable for carrier transfer due to the fact that the PbS QDs are electrically isolated from each other [14], which result in the decrease of the *J*_SC _values. Besides, the PbS QDs may limit the MEH-PPV infiltrate into the ZnO nanorod arrays. Because of the above effects, the exciton dissociation and charge carriers transfer efficiencies could be decreased, resulting in the reduction of the *η*_P_. Hence, the *η*_P _of most solar cells [13,24] with PbS QDs are not high although the PbS QDs have the wider absorption compared to other QDs. It indicates that it is possible to obtain high-efficiency hybrid solar cells using suitable QDs.

## Conclusions

In summary, an efficient PbS QD-sensitized MEH-PPV/ZnO nanorod hybrid solar cell was demonstrated. The 4 cycles of the PbS QD-sensitized solar cell showed a maximum *η*_P _of 0.42% under one sun illumination (AM1.5, 100 mW/cm^2^). The improved efficiency was attributed to the cascade structure formed by the PbS QD coating, which was unfavorable for carrier transfer after redundant coating. It was expected that by using the suitable nanostructures and QDs, the efficiency of the solar cells could be further improved.

## Competing interests

The authors declare that they have no competing interests.

## Authors' contributions

LW participated in the design of the study, carried out the experiments, collected data, performed data analysis, and drafted the manuscript. DZ and ZS participated in the design of the study and helped draft the manuscript. DS conceived the study, participated in its design, and helped draft the manuscript. All authors read and approved the final manuscript.
